# Identification of a Potential Dual-Target Candidate Against RSV F Protein and 15-LOX from TCMSP: Integrating Virtual Screening, Molecular Dynamics, and Experimental Evaluation

**DOI:** 10.3390/ijms27083448

**Published:** 2026-04-12

**Authors:** Xinyi Zhou, Haitao Du, Cheng Wang, Mengru Zhang, Xiaoyan Ding, Yi Wang, Zhonghao Fan, Ping Wang

**Affiliations:** 1 School of Pharmacy, Shandong University of Traditional Chinese Medicine, Jinan 250355, China; 15753332285@163.com (X.Z.); fzh01225@163.com (Z.F.); 2 Shandong Academy of Chinese Medicine, Jinan 250014, China; 306390275@163.com (C.W.); 15625156296@163.com (M.Z.); kate-66@163.com (X.D.); wyi_1989@163.com (Y.W.)

**Keywords:** dual-target inhibitor, respiratory syncytial virus, fusion glycoprotein, 15-lipoxygenase, high-throughput screening, molecular docking, molecular dynamics simulation

## Abstract

Respiratory syncytial virus (RSV) is a major pathogen responsible for severe lower respiratory tract infections in infants, the elderly, and immunocompromised individuals. Because the RSV F protein mediates viral entry and 15-lipoxygenase (15-LOX) amplifies virus-induced inflammatory responses, dual targeting of these proteins may provide both antiviral and anti-inflammatory benefits. In this study, we combined computational prediction with experimental validation to identify natural dual-target inhibitors from the Traditional Chinese Medicine Systems Pharmacology Database (TCMSP). A total of 13,131 natural compounds were screened by drug-likeness evaluation, molecular docking, ADME assessment, and molecular dynamics simulations, yielding 31 potential dual-target candidates with favorable drug-like properties. Among them, rhoeadine (MOL001473) maintained stable binding conformations with both targets throughout 100 ns simulations. In BEAS-2B cells, rhoeadine exhibited significant anti-RSV activity (EC50 = 1.82 µM), low cytotoxicity (IC50 = 34.50 µM), and a selectivity index (SI) of 18.97. Time-of-addition experiments suggested that rhoeadine primarily acts at the early stage of viral infection. Additionally, ELISA results indicated that rhoeadine significantly inhibited RSV-induced secretion of CCL5 and IL-6, highlighting its anti-inflammatory potential. In summary, this study identified rhoeadine as a promising natural compound with antiviral and anti-inflammatory activities against RSV. Computational analyses suggested its potential association with RSV F protein and 15-LOX, although direct target-level validation is still required.

## 1. Introduction

RSV is a leading pathogen responsible for severe lower respiratory tract infections in infants, young children, the elderly, and immunocompromised individuals [1]. During RSV infection, the viral F protein is regarded as the most critical and conserved structural protein—it directly mediates the fusion of the viral envelope with the host cell membrane, a mandatory step for viral entry [2]. To date, most approved or clinically developed RSV-specific antibodies and fusion inhibitors target the F protein. For instance, the next-generation long-acting antibody Nirsevimab (brand name Beyfortus^TM^) targets the prefusion conformation of the F protein and has been approved for the prevention of RSV in infants, demonstrating a favorable safety profile [3]. In the realm of fusion inhibitors, the small molecule GS-5806 (presatovir) locks the F protein in its prefusion state, thereby inhibiting viral entry, and has exhibited broad-spectrum anti-RSV activity in in vitro models [4], solidifying the central role of the F protein in anti-RSV interventions [5,6].

Concurrently, 15-LOX, a key rate-limiting enzyme in polyunsaturated fatty acid metabolism, plays a pivotal role in amplifying and sustaining inflammatory responses triggered by viral infections [7]. By catalyzing the production of leukotrienes and other inflammatory lipid mediators, 15-LOX serves as a crucial amplifier of airway inflammation and tissue damage associated with viral infections [8,9]. Studies in animal models have shown that RSV infection significantly upregulates 12/15-LOX expression in lung tissue and bronchoalveolar lavage fluid, concomitant with increased levels of inflammatory chemokines C-C Motif Chemokine Ligand 5 and C-C Motif Chemokine Ligand 3, which further exacerbate inflammatory responses and tissue injury [10]. Therefore, a dual-target strategy that concurrently inhibits both F protein and 15-LOX offers the potential for a synergistic “antiviral and anti-inflammatory” effect, while also potentially reducing the risk of drug resistance. This approach provides a theoretical foundation and an innovative direction for RSV therapy, aligning with the general principle of multi-target drug design: directly inhibiting the pathogen itself while modulating host-related pathways to halt disease progression [11].

In recent years, dual-target inhibitors have gained increasing attention as an emerging strategy in multifunctional drug design, offering the potential not only to enhance therapeutic efficacy but also to mitigate the common issue of resistance encountered in single-target therapies. For example, in the context of Type II diabetes, Juan P. Frias and colleagues developed the GLP-1/GIP dual receptor agonist Tirzepatide. A Phase III clinical trial comparing Tirzepatide with the single-target GLP-1 receptor agonist Semaglutide demonstrated that the dual-target agonist was significantly superior in reducing glycated hemoglobin (HbA1c) and body weight [12]. However, the screening of dual-target drugs faces the challenge of simultaneously satisfying the binding specificity and affinity for two structurally heterogeneous targets, a task difficult to accomplish on a large scale using traditional experimental methods alone [13]. Recent in silico studies have demonstrated the practical application of computer-aided drug design (CADD) in identifying bioactive compounds with potential therapeutic effects; for example, novel Cis-aconitate decarboxylase inhibitors were identified as potential anti-inflammatory agents using molecular docking and dynamics simulations [14]. In this context, computational chemistry methods provide a viable path for efficient and cost-effective preliminary screening [15]. Strategies such as high-throughput virtual screening, molecular docking, and MD simulations can rapidly identify potential candidate molecules from large compound libraries, predict their binding modes, stability, and dynamic behavior with target proteins, thereby offering robust support for subsequent experimental validation [16,17].

This study adopted an integrated computational-experimental strategy to identify and evaluate natural candidate compounds with potential relevance to both F protein and 15-LOX. We first conducted systematic virtual screening of the TCMSP, followed by molecular docking and MD simulations to prioritize natural small-molecule candidates with predicted high binding affinity and favorable drug-likeness. The top-ranked compound, rhoeadine, was then subjected to comprehensive in vitro validation. Its antiviral activity (EC_50_), cytotoxicity (IC_50_), selectivity index, time-of-addition profile, and suppressive effect on RSV-induced inflammatory cytokines were evaluated in human bronchial epithelial (BEAS-2B) cells. Together, these computational and experimental layers provide a coherent framework for identifying and characterizing a candidate compound with combined antiviral and anti-inflammatory properties and proposed dual-target relevance.

## 2. Results

### 2.1. Screening of Dual-Target Candidate Compounds

**Figure 1:**
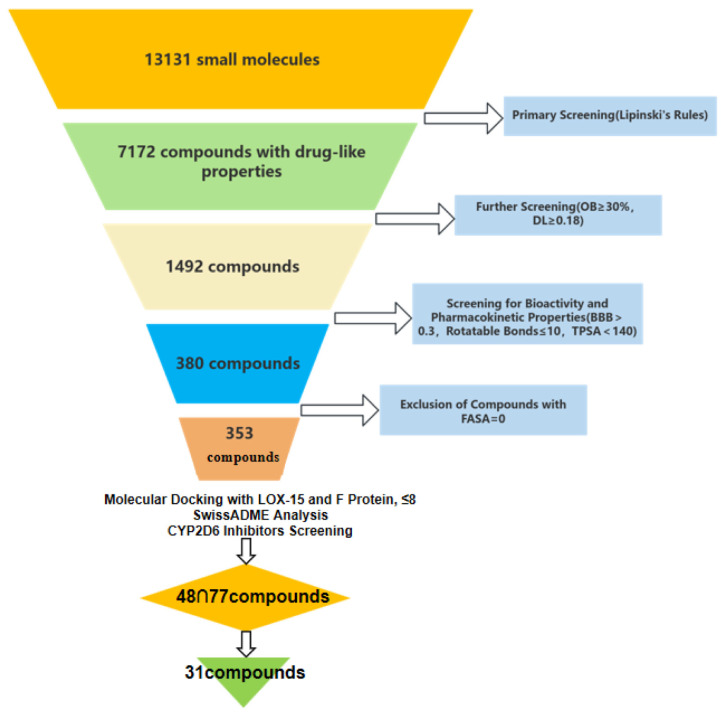
Flowchart of the small-molecule compound screening process.

To identify lead compounds from natural products that simultaneously target both 15-LOX and the F protein, a systematic high-throughput virtual screening of 13,131 small molecules from the TCMSP was performed (the workflow is illustrated in Figure 1). Based on the preliminary screening using Lipinski’s Rule of Five, 8492 small molecules were found to comply with the fundamental characteristics of oral drugs. Subsequent comprehensive evaluation, utilizing the parameters detailed in Section 4, yielded a final set of 353 structurally diverse candidate small molecules with favorable drug-like properties for subsequent molecular docking studies.

### 2.2. Quality Assessment of the Receptor Protein Structure Models

**Figure 2:**
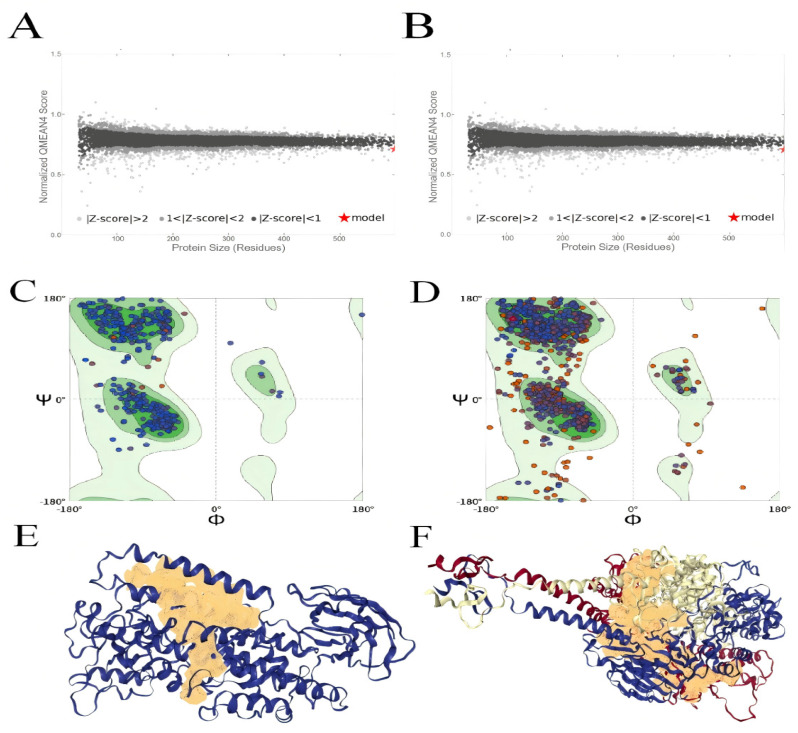
(**A**) QMEAN4 score plot for the 15-LOX model and (**B**) for the F protein model. The red asterisk indicates the QMEAN Z-score of the modeled structure relative to experimentally solved structures. (**C**) Ramachandran plot for the 15-LOX model and (**D**) for the F protein model. In panels (**C**,**D**), the colored background regions represent favored and allowed conformational regions of backbone dihedral angles, and each dot represents the φ/ψ angles of an individual amino acid residue. (**E**) Homology-modeled structure of 15-LOX and (**F**) of the F protein, depicting the predicted active sites. In panels (**E**,**F**), the protein backbone is shown as a blue cartoon representation, the predicted binding pocket/active-site region is highlighted in yellow surface representation, and the red arrows indicate the predicted active-site locations.

Homology modeling yielded a Global Model Quality Estimation (GMQE) of 0.91 for the 15-LOX model and 0.72 for the F protein model, both falling within the reliable range of 0 to 1. The Qualitative Model Energy Analysis (QMEAN) scores were −1.48 for 15-LOX and −1.55 for the F protein, situating within the expected range of −4 to 0. These results indicate a high degree of template-match reliability and overall model credibility for both structures. Furthermore, the QMEAN Z-scores (denoted by red asterisks) for both the 15-LOX (Figure 2A) and F protein (Figure 2B) models reside within the dark gray region (|Z-score| < 1), signifying considerable structural reliability and justifying their use in subsequent computational studies.

Validation using MolProbity yielded MolProbity scores of 1.71 and 1.61 for the 15-LOX and F protein models, respectively. Ramachandran plot analysis (Figure 2C,D) revealed that 95.14% of the residues in the 15-LOX model and 93.75% in the F protein model are located in the most favored regions. These outcomes collectively demonstrate high rationality and reliability in terms of geometric configuration, backbone conformation, and atomic spatial arrangement for both models.

To provide an intuitive visualization of the modeled spatial conformations and functionally relevant regions, the three-dimensional structures of 15-LOX and the F protein, along with their predicted active sites, are presented in Figure 2E and 2F, respectively. Distinct binding pockets were identified within the key regions of both the 15-LOX and F protein models. This observation is consistent with and corroborates the results from the GMQE, QMEAN, and Ramachandran plot analyses, thereby establishing a solid foundation for the subsequent molecular docking and molecular dynamics simulations.

### 2.3. Molecular Docking Results

**Figure 3:**
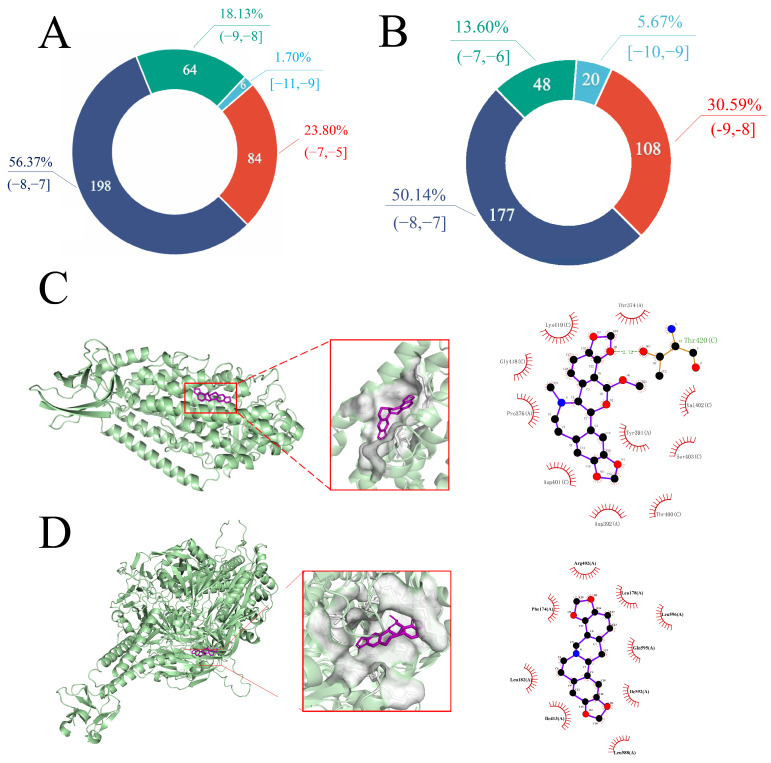
(**A**,**B**) Pie charts showing the distribution of molecular docking binding energies for (**A**) 15-LOX and (**B**) the F protein. The numerical values within the chart segments indicate the number and percentage of compounds falling within each specific binding energy range, as denoted in the legend. (**C**,**D**) Representative docking poses of the complexes between (**C**) 15-LOX and MOL001476, and (**D**) the F protein and MOL001473.

High-throughput molecular docking was performed against both the 15-LOX and the F protein using the 353 small-molecule compounds obtained from the aforementioned screening. The resulting binding affinity distributions are presented in Figure 3A and 3B, respectively. In the docking with 15-LOX, compounds such as (S)-Stylopine (MOL001476) and Cryptopin (MOL001460) exhibited the lowest binding energies (or highest binding affinities), indicating strong potential for interaction. For the F protein, molecules including rhoeadine and isocorynoline (MOL008640) ranked among the top candidates. The binding energy scores for all 353 molecules are detailed in Supplementary Tables S1 and S2.

Further analysis of representative docking poses revealed that these top-ranking molecules form stable interactions within the catalytic center of 15-LOX and the hydrophobic pocket adjacent to the fusion peptide of the F protein. Hydrophobic interactions and hydrogen bonding were identified as the key factors stabilizing these complexes and contributing to the high binding affinity. Representative docking poses for the MOL001476-15-LOX and MOL001473-F protein complexes are illustrated in Figure 3C and 3D, respectively.

### 2.4. Integrated Screening and Identification of Candidate Compounds

**Figure 4:**
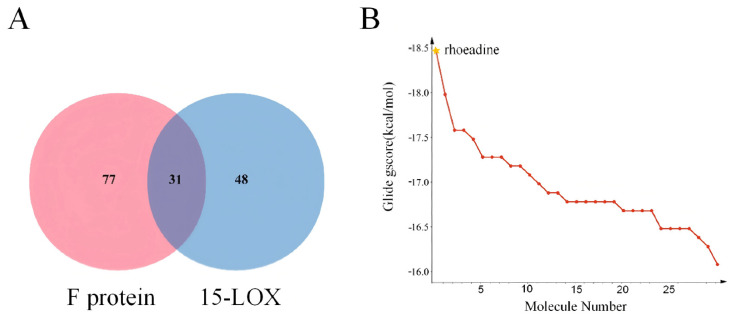
Comprehensive analysis of the candidate small-molecule compound screening results. Note: (**A**) Venn diagram illustrating the target activity overlap of candidate small molecules, identifying 31 compounds with potential activity against both the F protein and 15-LOX. (**B**) Line plot depicting the sum of binding energies for the 31 candidate compounds against the dual targets. The asterisk denotes rhoeadine, which exhibited the lowest cumulative binding energy.

To further identify small-molecule compounds with dual inhibitory activity, a systematic and integrated screening was conducted by combining the molecular docking results with ADME property predictions. As illustrated in the Venn diagram (Figure 4A), among the 353 candidate molecules, 31 compounds exhibited high binding affinity (binding energy ≤ −8 kcal/mol for both targets) and favorable ADME properties (Table 1), indicating potential dual-target inhibitory capability. As shown in the line chart (Figure 4B), among these 31 compounds, rhoeadine demonstrated the lowest sum of binding energies against both targets, coupled with satisfactory ADME properties. Therefore, it was prioritized as a representative candidate for subsequent molecular dynamics simulation and biological evaluation. This selection was based on its balanced overall performance in both predicted dual-target affinity and ADME-related characteristics.

The values listed in Table 1 were derived from two sources. The sum of dual-target binding energies was obtained from molecular docking and was used to reflect the overall predicted binding affinity of each compound toward both 15-LOX and the RSV F protein, with lower values indicating stronger predicted binding. The remaining parameters represent physicochemical and ADME-related descriptors collected during the drug-likeness screening and ADME evaluation processes. Specifically, mw, alogp, hdon, hacc, rbn, and TPSA were used to assess molecular properties relevant to drug-likeness; ob reflects predicted oral bioavailability; bbb indicates the predicted tendency for blood–brain barrier penetration; and dl and FASA are dimensionless descriptors used to support the evaluation of drug-likeness and molecular surface characteristics.

### 2.5. Molecular Dynamics Simulation Results

**Figure 5:**
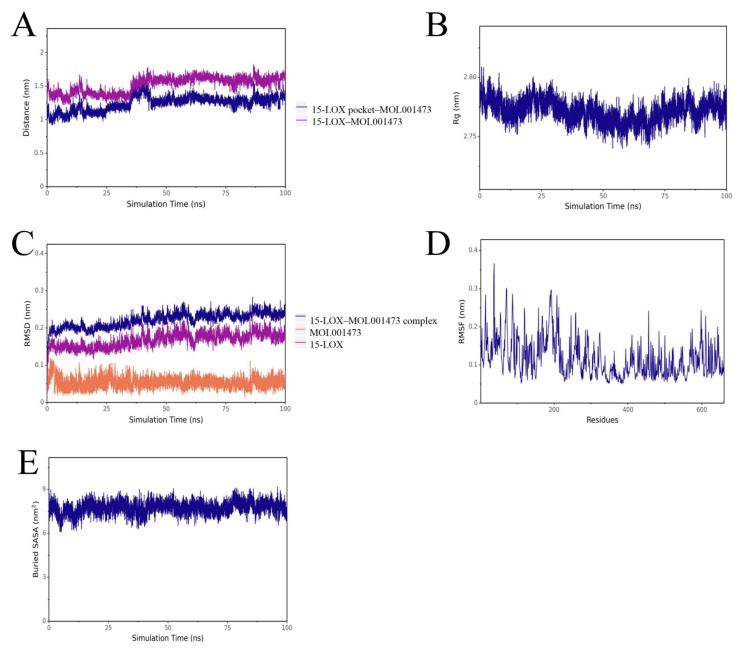
Molecular dynamics simulation profiles of the 15-LOX-MOL001473 complex. Note: (**A**) Distance between the protein binding site and the ligand. (**B**) Radius of gyration of the complex. (**C**) RMSD of the complex, protein, and ligand. (**D**) RMSF of the protein in the complex. (**E**) Buried solvent-accessible surface area is between the ligand and the protein.

**Figure 6:**
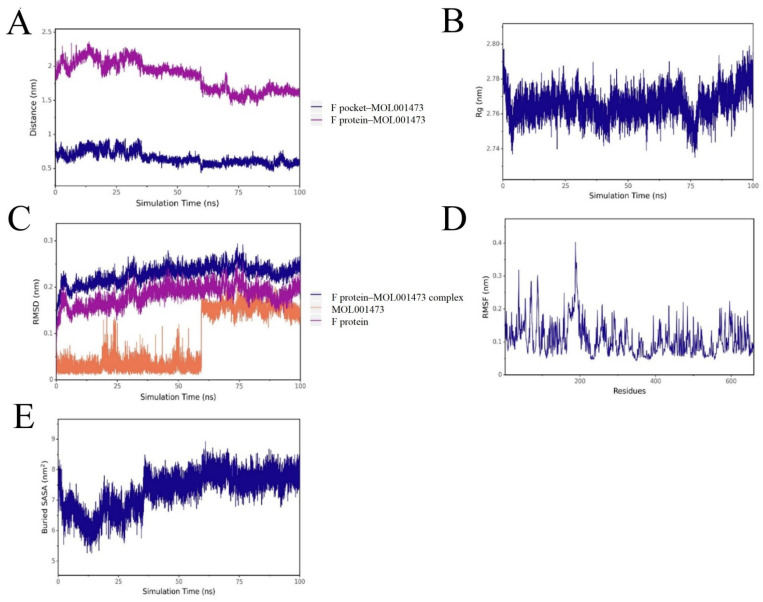
Molecular dynamics simulation profiles of the RSV F protein-MOL001473 complex. Note: (**A**) Distance between the protein binding site and the ligand. (**B**) Rg of the complex. (**C**) RMSD of the complex, protein, and ligand. (**D**) RMSF of the protein in the complex. (**E**) Buried SASA between the ligand and the protein.

Based on the high-throughput screening and molecular docking results, the most representative molecule, MOL001473, was selected for MD simulations with both 15-LOX and the RSV F protein. The results demonstrated high stability for both complexes throughout the simulations (Figure 5 and Figure 6). The root-mean-square deviation (RMSD) of the complexes exhibited minor fluctuations during the initial phase and subsequently plateaued, indicating that the systems reached conformational equilibrium after approximately 20 ns and remained stable thereafter. The radius of gyration (Rg) remained at stable levels during the entire 100 ns simulation, suggesting that ligand binding did not induce significant unfolding or global structural loosening in the proteins.

Analysis of the root-mean-square fluctuation (RMSF) revealed that protein flexibility was primarily concentrated in the surface loop regions, while residues in the vicinity of the binding pockets exhibited lower fluctuations. This further validates a stabilizing effect of the ligand on the key active sites. Results from the analysis of the distance between mass centers and the buried solvent-accessible surface area (Buried SASA) indicated that the small molecule maintained stable interactions within the binding pockets of both targets without significant dissociation. The contact area gradually stabilized during the later stages of the simulation.

Collectively, these results indicate that the candidate compound adopted and maintained a stable binding mode within the binding pockets of both 15-LOX and the F protein. These dynamic data further corroborate the binding poses predicted by molecular docking, thereby enhancing the reliability of Rhoeadine as a promising dual-target inhibitor.

### 2.6. Viral Titer

**Figure 7:**
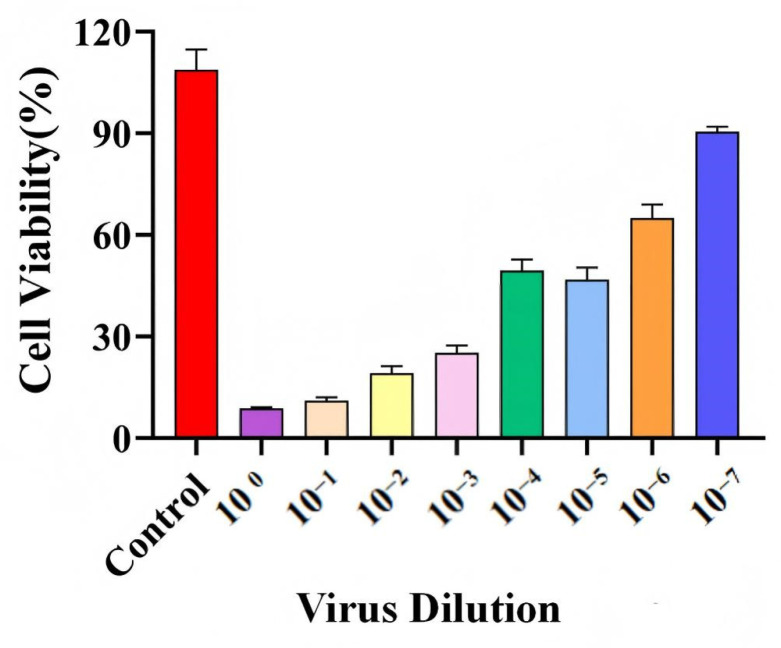
Cell viability of BEAS-2B cells following infection with serial dilutions of RSV strain A_2_. The corresponding numerical values (%) are as follows: Control (103.7), 10^0^ (8.7), 10^−1^ (11.1), 10^−2^ (19.1), 10^−3^ (25.2), 10^−4^ (49.4), 10^−5^ (46.7), 10^−6^ (65.0), and 10^−7^ (90.4).

The infectivity of RSV strain A_2_ was determined by viral titration in BEAS-2B cells using the TCID_50_ assay. As shown in Figure 7, the viability of BEAS-2B cells increased in a dose-dependent manner with increasing viral dilution. The infectious titer of the virus stock in BEAS-2B cells was calculated to be 3.97 log10 TCID_50_/mL by the Reed–Muench method. This titer was used in all subsequent infection experiments to ensure an inoculum of 100 TCID_50_ per well. These results demonstrate that RSV strain A_2_ possesses stable infectivity in vitro, thereby providing a reliable basis for viral inoculation in subsequent assays.

### 2.7. Half-Maximal Cytotoxic Concentration

**Figure 8:**
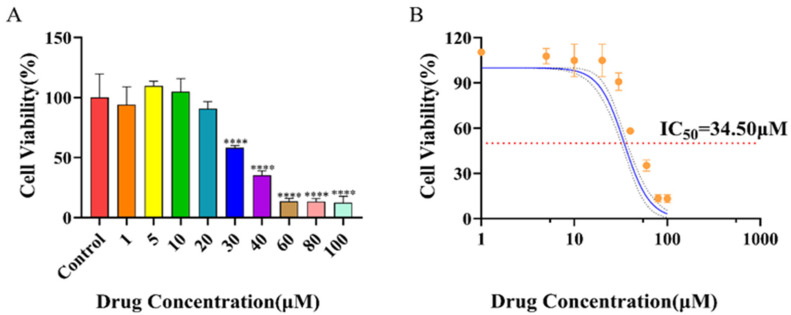
Cytotoxic effect of rhoeadine on BEAS-2B cells. Panel (**A**): The corresponding numerical values (%) are as follows: Control (100.0), 1 μM (94.2), 5 μM (109.7), 10 μM (105.0), 20 μM (90.8), 30 μM (58.2), 40 μM (35.2), 60 μM (13.6), 80 μM (13.3), and 100 μM (12.5). **** *p* < 0.0001. Panel (**B**): Dose–response curve of rhoeadine cytotoxicity. The solid line represents the nonlinear regression fit of the data, and the horizontal dashed line indicates the 50% cell viability threshold used to determine the IC_50_ value (34.50 µM). Cell viability (%) at each log_10_ concentration is as follows: log_10_ 0.000 (110.4), 0.699 (107.8), 1.000 (105.0), 1.301 (105.0), 1.477 (90.8), 1.602 (58.2), 1.778 (35.2), 1.903 (13.6), 2.000 (13.3). IC_50_ = 34.50 µM.

To evaluate the potential cytotoxicity of the candidate compound rhoeadine against host cells, its cytotoxic effect on BEAS-2B cells was assessed. As shown in Figure 8A, following 48 h of treatment with rhoeadine at concentrations ranging from 1 to 100 µM, cell viability decreased in a clear dose-dependent manner. Compared with the control group, a statistically significant reduction in cell viability was observed at concentrations ≥ 20 µM. Based on these data, the half-maximal cytotoxic concentration of rhoeadine was calculated to be 34.50 µM by fitting the dose–response curve (Figure 8B). These results indicate that rhoeadine exhibits no significant effect on cell growth at concentrations substantially lower than its IC_50_, thereby providing a broad and safe concentration window for subsequent antiviral efficacy studies.

### 2.8. Half-Maximal Effective Concentration and Selectivity Index

**Figure 9:**
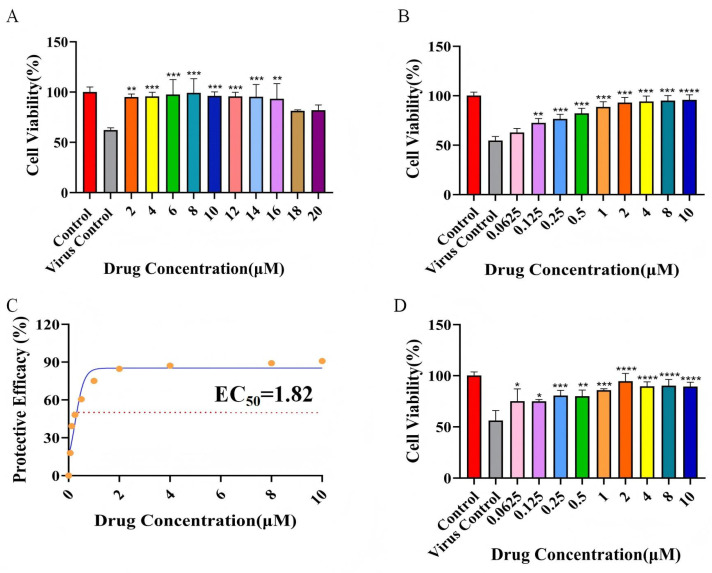
Anti-RSV activity and viral inhibition effect of rhoeadine. (**A**) Viability of BEAS-2B cells after 48 h of treatment with rhoeadine at concentrations ranging from 0 to 20 µM. (**B**) Viability of BEAS-2B cells after 48 h of treatment with rhoeadine at concentrations ranging from 0 to 10 µM. (**C**) Protection rate–concentration fitted curve; EC_50_ = 1.82 µM. (**D**) Cell viability at each serial dilution following inoculation of BEAS-2B cells with supernatants collected 48 h post-drug treatment. In panels (**A**,**B**,**D**), * *p* < 0.05, ** *p* < 0.01, *** *p* < 0.001, **** *p* < 0.0001 versus the untreated virus-infected control group. The corresponding numerical values (%) are summarized as follows: Panel (**A**): Control (100.0), Virus control (62.2), 2 μM (95.0), 4 μM (95.7), 6 μM (97.6), 8 μM (99.1), 10 μM (96.2), 12 μM (95.7), 14 μM (95.3), 16 μM (93.3), 18 μM (81.3), 20 μM (81.9). Panel (**B**): Control (100.2), Virus control (54.7), 0.0625 μM (62.9), 0.125 μM (72.5), 0.25 μM (76.5), 0.5 μM (82.2), 1 μM (88.7), 2 μM (93.1), 4 μM (94.2), 8 μM (95.1), 10 μM (95.8). Panel (**D**): Control (100.2), Virus control (56.2), 0.0625 μM (75.1), 0.125 μM (74.9), 0.25 μM (80.6), 0.5 μM (79.9), 1 μM (85.9), 2 μM (94.6), 4 μM (89.5), 8 μM (90.1), 10 μM (89.4).

To evaluate the anti-RSV activity of rhoeadine, preliminary testing was first conducted within a broad safe concentration range (0–20 µM). Cell viability under these conditions is shown in Figure 9A. Calculation of the cytoprotection rate revealed that the protection values varied within a narrow range across this concentration interval, precluding an accurate determination of the EC_50_. Therefore, the experimental design was optimized, and the formal assay was performed using an adjusted concentration range of 0–10 µM; cell viability data are presented in Figure 9B. The protection rate–concentration curve was calculated and fitted (Figure 9C), yielding an EC_50_ value of 1.82 µM for rhoeadine against RSV. Combined with the IC_50_ value of 34.50 µM obtained from the cytotoxicity assay, the selectivity index of rhoeadine was calculated to be 18.97. An SI greater than 10 indicates that rhoeadine possesses a clear antiviral therapeutic window in vitro.

To directly assess the effect of rhoeadine on RSV replication, cell culture supernatants were collected 48 h post-treatment with various concentrations of the compound, and the infectious viral titers in the supernatants were determined by the TCID_50_ assay. Across all treatment groups (0–10 µM), following serial dilution and inoculation onto BEAS-2B cells, cell viability at each dilution remained above 70% (Figure 9D). This observation indicates that the content of infectious viral particles in the supernatants was substantially reduced after rhoeadine treatment. These virological findings demonstrate that rhoeadine potently inhibits RSV replication, which is in strong agreement with its pronounced cytoprotective effects observed at the cellular level.

### 2.9. Results of the Time-of-Addition Assay

**Figure 10:**
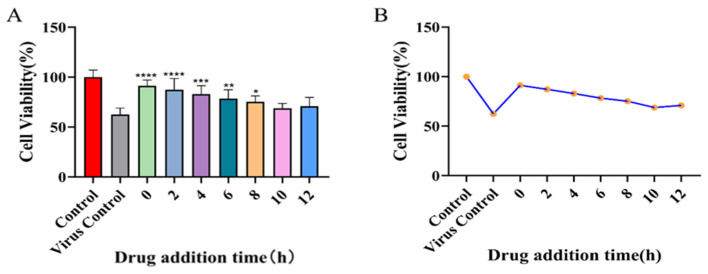
Effect of time of drug addition on the anti-RSV activity of rhoeadine. Note: * *p* < 0.05, ** *p* < 0.01, *** *p* < 0.001, **** *p* < 0.0001 versus the virus control group. Panel (**A**): The corresponding numerical values (%) are as follows: Control (100.0), Virus control (62.5), 0 h (91.2), 2 h (87.2), 4 h (82.9), 6 h (78.4), 8 h (75.2), 10 h (68.9), and 12 h (71.0). Panel (**B**): The corresponding numerical values (%) are as follows: Control (100.0), Virus control (62.5), 0 h (91.2), 2 h (87.2), 4 h (82.9), 6 h (78.4), 8 h (75.2), 10 h (68.9), and 12 h (71.0).

To preliminarily delineate the stage of the viral life cycle targeted by the antiviral activity of rhoeadine, BEAS-2B cells were infected with RSV, and 10 µM of the compound was added at various time points post-infection. Cell viability was assessed at 48 h post-infection. The results demonstrated that the most pronounced cytoprotective effect of rhoeadine was observed when the compound was administered concurrently with viral adsorption (0 h) or during the early phase of infection (2–4 h post-infection), as evidenced by significantly higher cell viability compared with the virus control group. However, when drug addition was delayed until 8 h post-infection or later, the protective effect was markedly diminished (Figure 10). These findings suggest that rhoeadine exerts its antiviral activity primarily by interfering with early stages of the viral life cycle, such as viral adsorption or entry, a mode of action consistent with that of typical RSV fusion protein inhibitors.

### 2.10. Inflammatory Cytokine Detection Results

**Figure 11:**
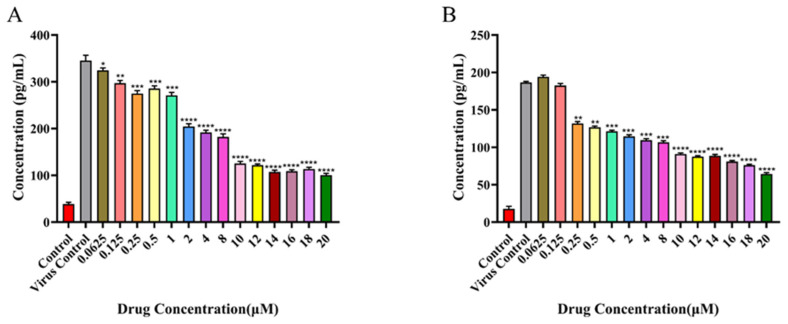
Inhibitory effect of rhoeadine on the release of inflammatory cytokines induced by RSV infection. Note: (**A**) Effect of different concentrations of rhoeadine on CCL5/RANTES secretion following RSV infection; (**B**) Effect of different concentrations of rhoeadine on IL-6 secretion following RSV infection. * *p* < 0.05, ** *p* < 0.01, *** *p* < 0.001, **** *p* < 0.0001 versus the virus control group. The corresponding numerical values (%) are summarized as follows: Panel (**A**) (CCL5): Control (38.5), Virus control (345.3), 0.0625 μM (324.3), 0.125 μM (296.9), 0.25 μM (274.8), 0.5 μM (285.5), 1 μM (270.8), 2 μM (204.5), 4 μM (191.8), 8 μM (182.0), 10 μM (125.1), 12 μM (121.6), 14 μM (107.3), 16 μM (108.8), 18 μM (113.5), and 20 μM (100.5). Panel (**B**) (IL-6): Control (17.6), Virus control (186.5), 0.0625 μM (194.1), 0.125 μM (182.5), 0.25 μM (131.9), 0.5 μM (126.5), 1 μM (121.3), 2 μM (114.5), 4 μM (109.5), 8 μM (106.5), 10 μM (90.9), 12 μM (87.4), 14 μM (88.6), 16 μM (80.9), 18 μM (76.1), and 20 μM (64.2).

To evaluate the effect of rhoeadine on the inflammatory response induced by RSV infection, the secretion levels of the inflammatory cytokines CCL5/RANTES and IL-6 in the infected supernatants of BEAS-2B cells were measured by ELISA. The results showed that RSV infection significantly promoted the release of CCL5 and IL-6; compared with the normal cell control group, the levels of these inflammatory cytokines in the virus control group were increased by approximately 3.5 times and 4.2 times, respectively. Following treatment with various concentrations of rhoeadine, the secretion of both cytokines decreased in a dose-dependent manner. At a concentration of 10 µM, the secretion levels of CCL5 and IL-6 were markedly reduced compared with those in the virus control group (Figure 11A,B). These findings indicate that rhoeadine not only possesses antiviral activity but also effectively inhibits the release of inflammatory cytokines induced by RSV infection, suggesting that it may exert an adjunctive therapeutic effect through modulation of the host inflammatory response.

## 3. Discussion

Current clinical interventions for RSV still rely predominantly on single-target strategies, particularly fusion inhibitors or monoclonal antibodies directed against the viral F protein. For instance, nirsevimab has been shown to significantly reduce hospitalization rates due to RSV-associated lower respiratory tract infections in infants in clinical studies [18,19], while GS-5806 demonstrated considerable antiviral activity in adult infection models [20]. Although these strategies have achieved success in blocking viral entry, they may be vulnerable to resistance arising from viral mutations and do not directly address the inflammatory injury triggered by excessive host immune activation during disease progression. 15-LOX, a key enzyme in the synthesis of inflammatory lipid mediators, has been confirmed to be closely associated with airway hyper-inflammatory responses in RSV infection models [21]. Elevated levels of its products have also been observed in COVID-19 patients, suggesting its universal role in amplifying inflammation in viral infections [22]. Therefore, this study explored the possibility that simultaneous modulation of 15-LOX-related inflammatory pathways and RSV F protein-associated viral entry may contribute to combined antiviral and anti-inflammatory effects. This approach aligns with the emerging trend in multi-target drug development for complex diseases such as diabetes, cancer, and neurodegenerative disorders. Compared with conventional single-target RSV therapies, this strategy may provide a broader therapeutic profile by combining antiviral activity with modulation of host inflammatory responses.

This study employed an integrated strategy combining computational prediction and experimental evaluation to explore the feasibility of a dual-target hypothesis against RSV. Through computational screening, MOL001473 was identified from a natural product library as a potential dual-target candidate molecule. As shown in Figure 3, only a limited number of compounds maintained favorable predicted interactions with both 15-LOX and the RSV F protein simultaneously. Further integrated analysis of Figure 4 and Table 1 showed that rhoeadine exhibited the lowest summed binding energy toward the two targets while also retaining acceptable physicochemical and ADME-related properties, supporting its prioritization for subsequent validation. Subsequent in vitro experiments provided phenotypic support for these predictions: rhoeadine demonstrated anti-RSV activity in the BEAS-2B cell model, thereby preliminarily supporting its computationally predicted biological potential at the cellular level.

Mechanistic investigations revealed that rhoeadine possesses distinct target-directed properties. Time-of-addition assays demonstrated that its cytoprotective effect was predominantly confined to the early stages of viral infection, especially during viral adsorption and within 2–4 h post-infection, which is consistent with the characteristic time frame of action of F protein inhibitors. Furthermore, the compound concentration-dependently suppressed RSV infection-induced secretion of CCL5 and IL-6, supporting its anti-inflammatory activity, which is consistent with the predicted involvement of 15-LOX-related pathways. These findings preliminarily support, at the cellular phenotypic level, the antiviral and anti-inflammatory profile of rhoeadine, while direct target-level validation remains necessary. In addition, rhoeadine exhibited measurable antiviral efficacy in BEAS-2B cells, with an EC50 of 1.82 µM, an IC50 of 34.50 µM, and a selectivity index of 18.97. Taken together, these findings preliminarily support the dual antiviral and anti-inflammatory pharmacological profile of rhoeadine at the cellular level.

Nevertheless, direct biochemical validation of 15-LOX inhibition and direct binding verification toward the RSV F protein are still needed. In addition, the current findings were obtained in a single in vitro model, and further validation in other experimental systems and in vivo models will be necessary.

## 4. Materials and Methods

### 4.1. Compound Library Acquisition

All small-molecule compounds utilized in this study were sourced from the TCMSP (https://www.tcmsp-e.com/). This database systematically compiles the structures, targets, and ADME properties of active ingredients derived from Chinese herbs, serving as a core resource for modern research in traditional medicine [23]. We comprehensively retrieved the raw structural data (in mol2 format) of all 13,131 small-molecule compounds contained within TCMSP (accessed on 20 May 2025). No filtration criteria were applied during the download process to preserve the integrity and structural diversity of the compound library.

### 4.2. High-Throughput Virtual Screening

The initial library of 13,131 small-molecule compounds obtained from the TCMSP was subjected to a preliminary filtration based on Lipinski’s Rule of Five (molecular weight < 500, number of hydrogen bond donors < 5, number of hydrogen bond acceptors < 10, and octanol-water partition coefficient log P < 5) to exclude compounds that violated these fundamental principles of drug-likeness [24]. Subsequently, oral bioavailability (OB ≥ 30%) and drug-likeness (DL ≥ 0.18), as defined in the TCMSP User Guide, were applied as key screening criteria to evaluate the compounds’ potential for in vivo absorption and their pharmaceutical properties [25]. Further refinement of the compound set was performed based on blood–brain barrier permeability (BBB < 0.3), the number of rotatable bonds (≤10), and topological polar surface area (TPSA < 140) to assess their bioactivity and pharmacokinetic profiles. Additionally, compounds with a zero fractional accessible surface area (FASA = 0) were excluded [26,27]. The resulting subset of small-molecule compounds from this multi-step screening pipeline was retained for subsequent molecular docking studies to further investigate their binding affinity and mechanism of action with the target proteins.

### 4.3. Acquisition and Modeling of Receptor Protein Structures

The amino acid sequence of human 15-lipoxygenase (UniProt ID: P16050) was retrieved from the UniProt database (https://www.uniprot.org/) [28]. The reference structure of the RSV fusion glycoprotein (PDB ID: 5EA5) with a resolution of 3.05 Å was obtained from the RCSB Protein Data Bank (https://www.rcsb.org/). Three-dimensional models for both targets were subsequently constructed using the SWISS-MODEL homology modeling server (https://swissmodel.expasy.org/) [29], selecting the template with the highest sequence similarity for each.

Following model construction, the quality of the generated models was assessed using parameters including GMQE and QMEAN. Geometric structure and backbone conformation were further analyzed, with the MolProbity score and Ramachandran plots serving as additional validation metrics. Active sites were predicted using the ProteinsPlus server (https://proteins.plus/) [30]. The final models, incorporating the predicted active site information, were prepared with domain coordinate files and exported in PDB format for subsequent molecular docking studies.

### 4.4. Molecular Docking

#### 4.4.1. Preparation of Receptors and Ligands

The protein structures were prepared using PyMOL (version 2.3.4) to remove water molecules and extraneous ligands. Hydrogen atoms were added, and energy minimization was performed to optimize the charge distribution. The processed receptor structures were subsequently saved in PDBQT format for docking [31].

The small-molecule compounds, initially obtained in MOL2 format from the TCMSP, were converted into PDBQT format using OpenBabel software (version 3.1.1).

#### 4.4.2. Docking Parameter Configuration

Based on the active sites predicted by the ProteinsPlus server, the catalytic pocket center for 15-LOX was defined at coordinates (x = −28.86, y = 149.45, z = 53.09) with a grid box of maximum radius 26.79 Å. For the RSV F protein, the binding site center was set at coordinates (x = −18.10, y = −15.20, z = −41.67) with a maximum radius of 40.78 Å. Molecular docking was performed using AutoDock Vina, with all other parameters maintained at their default settings. The grid center and search space were defined based on the active-site regions predicted by the ProteinsPlus server and were set to fully cover the expected binding pockets of both targets. In addition, the top-ranked docking poses were further inspected to confirm that they were located within reasonable binding regions and formed plausible interactions with surrounding key residues. This procedure was used to support the suitability of the docking parameter settings for subsequent virtual screening.

#### 4.4.3. Molecular Docking Execution

The preprocessed receptor protein and ligand files, along with the configured docking parameters, were subjected to molecular docking calculations using AutoDock Vina 1.2.0. This process simulated the binding conformations of the ligand molecules within the active sites of the respective receptors. The calculated binding affinity values were recorded for each complex. The resulting docking poses were visualized and analyzed using PyMOL and LigPlot+ (version 2.2.9) [32]. All data generated from the molecular docking simulations were systematically archived and served as the evaluation basis for the subsequent screening of dual-target compounds.

### 4.5. Comprehensive Screening Integrating Molecular Docking and ADME Analysis

A binding affinity threshold of <−8 kcal/mol, as calculated by molecular docking, was applied to evaluate the binding strength of the compounds to the target proteins [33]. Compounds meeting this criterion were subsequently subjected to ADME analysis using the SwissADME web server (http://www.swissadme.ch/ accessed on 20 May 2025) [34]. This integrated approach enabled the identification of small-molecule compounds with potential dual-target inhibitory activity, thereby establishing a foundation for subsequent molecular dynamics simulations.

### 4.6. Molecular Dynamics Simulations

MD simulations were performed using the GROMACS 2022 software package. The GAFF force field was applied to the small molecules, while the AMBER14SB force field and the TIP3P water model were used for the protein and the solvent, respectively. The protein and ligand structures were integrated to construct the simulation system for the complex. Simulations were conducted under isothermal-isobaric (NPT) ensemble conditions with periodic boundary constraints. During the simulations, all bonds involving hydrogen atoms were constrained using the LINCS algorithm, allowing for an integration time step of 2 fs. Electrostatic interactions were calculated using the Particle Mesh Ewald (PME) method with a cutoff of 1.2 nm. The non-bonded interaction cutoff was set to 10 Å and updated every 10 steps. Temperature was maintained at 298 K using the V-rescale thermostat, and pressure was controlled at 1 bar using the Berendsen barostat. The system underwent equilibration for 100 ps each under canonical (NVT) and isothermal-isobaric ensembles at 298 K, followed by a production MD run of 100 ns [35]. Trajectory frames were saved every 10 ps for subsequent analysis. Following the simulation, the resulting trajectories were analyzed using VMD 1.9.3 and PyMOL 2.6.2.

### 4.7. In Vitro Biological Activity

#### 4.7.1. Reagents and Consumables

Rhoeadine (Pin Ke Yan, Hong Kong, China, Cat: MB6979A); high-glucose DMEM (Gibco, Waltham, MA, USA, Cat: 2353429); 0.25% trypsin (NCM Biotech, Newport, RI, USA, Cat: C100C1); penicillin-streptomycin solution (Beyotime, Shanghai, China, Cat: C0222); fetal bovine serum (ExCell Bio, Shanghai, China, Cat: 12B234); cell culture-grade dimethyl sulfoxide (Solarbio, Beijing, China, Cat: D8370); human CCL5 ELISA kit (Enzyme-linked Biotechnology, Shanghai, China, Cat: ml037928); and human IL-6 ELISA kit (Enzyme-linked Biotechnology, Cat: ml058097). A microplate spectrophotometer (Model SpectraMax i3x, Manufacturer Molecular Devices, San Jose, CA, USA) was used to measure absorbance at 450 nm in the CCK-8-based cell viability and viral titer assays.

#### 4.7.2. Preparation of Compound Stock Solution

Rhoeadine was first dissolved in cell culture-grade dimethyl sulfoxide (DMSO) to prepare a stock solution and was subsequently diluted with culture medium to the indicated working concentrations immediately before use. In all in vitro biological assays, the final concentration of DMSO was kept constant across treatment and control groups and maintained at a level that did not affect cell viability. The same solvent-control principle was applied in the cytotoxicity assay, antiviral activity assay, time-of-drug administration assay, and cytokine determination experiments to ensure experimental reproducibility.

#### 4.7.3. Cell Lines and Viruses

The human lung epithelial cell line and respiratory syncytial virus (RSV, strain A2) were used in this study. BEAS-2B cells were purchased from Shanghai Celliver Biotechnology Co., Ltd. (Shanghai, China, Cat: STCC10202G-1). Prior to use, the cells were authenticated by short tandem repeat (STR) analysis and confirmed to be free of mycoplasma contamination. RSV strain A2 was provided and preserved by the Institute of Basic Medicine, Shandong Academy of Medical Sciences. Before experiments, the virus was propagated in BEAS-2B cells and titrated to determine its infectious titer; working stocks of the virus were stored at –80 °C.

#### 4.7.4. Determination of Viral Titer

BEAS-2B cells were seeded into 96-well plates at a density of 2.5 × 104 cells per well and cultured until reaching >90% confluence prior to viral infection. The virus stock was serially diluted 10-fold with serum-free medium to yield eight concentrations (100 to 10^−7^). Each dilution was added to the cells at 100 µL per well, with six replicate wells per dilution. Uninfected cell controls and blank wells containing only culture medium were also included. The plates were incubated at 37 °C in a humidified atmosphere with 5% CO_2_ for 48 h. After infection, CCK-8 reagent was added to each well and incubated for 1 h, and the absorbance at 450 nm was measured using a microplate reader. Cell viability was calculated by comparing the absorbance values of experimental wells with those of blank controls. The viral titer, expressed as lg TCID_50_/mL, was determined using the Reed–Muench method.

#### 4.7.5. Cytotoxicity Assay

BEAS-2B cells were seeded into 96-well plates at a density of 2.5 × 10^4^ cells per well. Upon reaching >90% confluence, the culture medium was replaced with fresh medium containing a series of concentrations (1, 5, 10, 20, 30, 40, 60, 80, 100 µM) of the test compound, and the cells were incubated for an additional 48 h. Six replicate wells were set for each concentration, and a cell control group without drug treatment was included. After incubation, CCK-8 solution was added to each well and incubated; the absorbance at 450 nm was then measured using a microplate reader. Cell viability was subsequently calculated, and the dose–response curve was fitted to determine the half-maximal cytotoxic concentration value.

#### 4.7.6. Antiviral Activity Assay

To determine the antiviral activity of rhoeadine, preliminary screening was performed within a previously established safe concentration range (0–20 µM). Based on the screening results, a series of concentrations (0.0625, 0.125, 0.25, 0.5, 1, 2, 4, 8, and 10 µM) was selected for the formal assay to precisely quantify its potency. BEAS-2B cells were incubated with 100 TCID_50_ of RSV strain A2 for 2 h, after which the medium was replaced with maintenance medium containing the corresponding concentrations of the test compound. The cells were then cultured for an additional 48 h. Six replicate wells were set for each concentration, and both virus controls and cell controls were included.

##### Supernatant Collection

The supernatant from each well was carefully aspirated and aliquoted into two microcentrifuge tubes: one portion was used for viral titer determination by the TCID_50_ assay, and the other was subjected to ELISA for the detection of inflammatory cytokines.

##### Determination of Viral Titer in Supernatants

The residual infectious viral particles in the supernatants following drug treatment were quantified using the TCID_50_ assay. The collected supernatants were serially diluted 10-fold (10^−1^ to 10^−7^) with serum-free DMEM medium. Each dilution was added to 96-well plates seeded with BEAS-2B cells at 100 μL per well, with six replicate wells per dilution. Uninfected cell controls were also included. Subsequent procedures were carried out as described in Section 4.7.4, and the TCID_50_ titers of the viruses in the supernatants were calculated using the Reed–Muench method. The results were expressed as lg TCID_50_/mL.

##### Cell Viability Assay

CCK-8 reagent was added to the original wells described in Section 4.7.6 that had been subjected to drug treatment and viral infection. After incubation for 1 h, the absorbance was measured at 450 nm. Cell viability and the inhibition rate of virus-induced cytopathic effect were calculated. The dose–response curve was then fitted to determine the half-maximal effective concentration of the compound, and the selectivity index was subsequently calculated.

#### 4.7.7. Time-of-Drug Administration Assay

BEAS-2B cells cultured in 96-well plates were infected with 100 TCID_50_ of RSV strain A2 for 2 h. At 0 h (co-treatment), 2, 4, 6, 8, 10, and 12 h post-infection, the medium was replaced with maintenance medium containing 10 µM rhoeadine. In the co-treatment group (0 h), the compound was added simultaneously with viral inoculation. At 48 h post-infection, cell viability was assessed using the CCK-8 assay. Cell viability was then plotted against the time of drug addition to generate a time-of-addition response curve.

#### 4.7.8. Determination of Inflammatory Cytokines

Following the completion of the antiviral pharmacodynamic assays and collection of the infected BEAS-2B cell supernatants, the concentrations of the corresponding inflammatory cytokines in the cell supernatants harvested in Section 4.7.6 were determined using human CCL5/RANTES and human IL-6 ELISA kits, strictly according to the manufacturers’ instructions. The anti-inflammatory activity of the compound was evaluated by comparing the levels of inflammatory cytokine secretion between the drug-treated groups and the virus-infected control group.

#### 4.7.9. Statistical Analysis

All experiments were independently performed in triplicate, and the data are expressed as mean ± standard deviation (SD). Statistical significance was set at * *p* < 0.05, ** *p* < 0.01, *** *p* < 0.001, and **** *p* < 0.0001. Cell viability was calculated using the following formula: Cell viability (%) = OD_450_ (treated) − OD_450_ (blank)/OD450 (control) − OD_450_ (blank) × 100%. The protection rate was calculated as: Protection rate (%) = OD_450_ (drug-treated) − OD_450_ (virus control)/OD_450_ (cell control) − OD_450_ (virus control) × 100%. The selectivity index was determined using the equation: SI = IC_50_/EC_50_. Comparisons among groups were performed by one-way analysis of variance (ANOVA). All statistical analyses and graphical presentations were conducted using GraphPad Prism software (version 10.1.2).

## 5. Conclusions

In this study, we established an integrated computational–experimental workflow to identify candidate natural compounds with antiviral and anti-inflammatory potential against RSV from the TCMSP natural-product library. Virtual screening, molecular docking, ADME evaluation, and 100 ns molecular dynamics simulations prioritized rhoeadine as a candidate compound with favorable predicted interactions with both targets. In BEAS-2B cells, rhoeadine exhibited potent anti-RSV activity (EC_50_ = 1.819 μM) with low cytotoxicity (IC_50_ = 34.50 μM) and a favorable selectivity index (SI = 18.97). Time-of-addition assays indicated that rhoeadine primarily interferes with early stages of infection, consistent with an RSV entry/fusion-related mechanism, while ELISA data showed significant suppression of RSV-induced CCL5 and IL-6 secretion, supporting its anti-inflammatory potential. Collectively, these findings support rhoeadine as a promising natural compound with antiviral and anti-inflammatory activities against RSV and warrant further target-level validation and mechanistic characterization.

## Figures and Tables

**Table 1:** List of 31 candidate small molecules with high binding potential for both targets.

Molecule Number	MOL ID	The Sum of Dual-Target Binding Energies	Molecule Name	mw	aLogp	hdon	hacc	CYP2D6 Inhibitor	ob	bbb	dl	FASA	rbn	tpsa
1	MOL001473	−18.4	rhoeadine	383.43	2.847	0	7	Yes	63.51407829	0.46958	0.83082	0.22185527	1	58.62
2	MOL001476	−17.9	(S)-Stylopine	323.37	3.201	0	5	Yes	51.14580282	0.50254	0.85472	0.26929522	0	40.16
3	MOL004891	−17.5	shinpterocarpin	322.38	3.461	1	4	Yes	80.29527688	0.68497	0.72746	0.31749722	0	47.92
4	MOL004230	−17.5	stylopine	323.37	3.201	0	5	Yes	48.24918701	0.51696	0.85474	0.27183107	0	40.16
5	MOL002862	−17.4	pipercide	353.5	4.782	1	4	Yes	42.72282872	0.45829	0.43092	0.34501058	9	47.56
6	MOL001462	−17.2	Dihydrochelirubine	363.39	3.692	0	6	Yes	55.2942536	0.36515	0.85677	0.24188675	1	49.39
7	MOL006971	−17.2	CREBANINE	339.42	3.335	0	5	Yes	34.63777605	0.62196	0.74554	0.21267189	2	40.16
8	MOL012216	−17.2	norlobelanine	321.45	3.767	1	3	Yes	64.07963067	0.32689	0.29764	0.37672523	6	46.17
9	MOL001614	−17.1	(E,E,E)-11-(1,3-Benzodioxol-5-yl)-N-(2-methylpropyl)-2,4,10-undecatrienenamide	353.5	4.782	1	4	Yes	42.72282872	0.34472	0.43055	0.34429699	9	47.56
10	MOL007036	−17.1	5,6-dihydroxy-7-isopropyl-1,1-dimethyl-2,3-dihydrophenanthren-4-one	298.41	4.377	2	3	Yes	33.76525236	0.80305	0.28585	0.29250512	1	57.53
11	MOL007218	−17.0	Remerin	279.36	3.368	0	3	Yes	40.75491578	0.97056	0.5208	0.31650063	0	21.7
12	MOL007985	−16.9	austrobailignan-5	326.4	4.961	0	4	Yes	42.54318497	0.41811	0.37535	0.34001276	5	36.92
13	MOL002309	−16.8	indirubin	262.28	1.877	2	4	Yes	48.59034694	0.44414	0.25929	0.43639734	0	65.72
14	MOL005003	−16.8	Licoagrocarpin	338.43	4.514	1	4	Yes	58.81390287	0.60553	0.58498	0.27100939	3	47.92
15	MOL002605	−16.7	11-Hydroxynumantenine	370.49	1.575	1	6	Yes	50.79488717	0.42079	0.70642	0.26286873	1	62.24
16	MOL001454	−16.7	berberine	336.39	3.447	0	4	Yes	36.86124504	0.56718	0.77665	0.19133656	2	40.8
17	MOL005405	−16.7	Cusparine	307.37	4.206	0	4	Yes	68.1632046	0.36786	0.392	0.32831806	4	40.58
18	MOL007101	−16.7	Dihydrotanshinone I	278.32	2.858	0	3	Yes	45.04327919	0.42535	0.36015	0.39671803	0	43.37
19	MOL009149	−16.7	Cheilanthifoline	325.39	3.15	1	5	Yes	46.50503762	0.52073	0.72251	0.24662723	1	51.16
20	MOL000793	−16.7	C09367	325.39	3.084	1	5	Yes	47.5379008	0.70311	0.69308	0.24708159	1	51.16
21	MOL002991	−16.6	(6aR,11aR)-3,9-dimethoxy-6a,11a-dihydro-6H-benzofurano[3,2-c]chromene-4,10-diol	316.33	2.374	2	6	Yes	38.96099145	0.35403	0.48083	0.21266416	2	77.38
22	MOL001484	−16.6	Inermine	284.28	2.442	1	5	Yes	75.18306038	0.39505	0.53754	0.29992098	0	57.15
23	MOL003956	−16.6	dihydrorutaecarpine	289.36	3.314	2	3	Yes	42.26686143	0.69793	0.59633	0.31565556	0	48.13
24	MOL011912	−16.6	(2R,4aS,10aR)-7-isopropyl-2,4a-dimethyl-1-methylene-4,9,10,10a-tetrahydro-3H-phenanthren-2-ol	284.48	4.947	1	1	Yes	48.60937084	1.14295	0.25123	0.29044822	1	20.23
25	MOL000456	−16.4	Phaseolin	322.38	3.461	1	4	Yes	78.20058465	0.38953	0.72891	0.33217204	0	47.92
26	MOL003648	−16.4	Inermin	284.28	2.442	1	5	Yes	65.83093145	0.36121	0.53754	0.30202371	0	57.15
27	MOL001560	−16.4	pipernonaline	341.49	4.69	0	4	Yes	51.31566957	0.33249	0.4092	0.29390231	7	38.77
28	MOL008457	−16.4	Tetrahydroalstonine	352.47	2.664	1	4	Yes	32.41977527	0.32735	0.81311	0.23339006	2	54.56
29	MOL010495	−16.3	6,7-dimethoxy-2-(2-phenylethyl)chromone	310.37	3.881	0	4	Yes	31.92860911	0.37057	0.29833	0.30336142	5	48.67
30	MOL004193	−16.2	Clarkeanidine	327.41	3.098	2	5	Yes	86.6502859	0.4161	0.53556	0.21372631	2	62.16
31	MOL008633	−16.0	(S)-Cheilanthifoline	325.39	3.15	1	5	Yes	46.68205866	0.43576	0.72434	0.22936389	1	51.16

Note: The sum of dual-target binding energies is expressed in kcal/mol; mw in Da; tpsa in Å^2^; ob in %; hdon, hacc, and rbn are counts; aLogp, bbb, dl, and FASA are dimensionless descriptors and predicted parameters.

## Data Availability

The original contributions presented in this study are included in the article/Supplementary Material. Further inquiries can be directed to the corresponding authors.

## Supplementary Materials

The following supporting information can be downloaded at: https://www.mdpi.com/article/10.3390/ijms27083448/s1.

## Abbreviations

The following abbreviations are used in this manuscript:

RSV	Respiratory Syncytial Virus
F protein	Fusion Protein
15-LOX	15-lipoxygenase
TCMSP	Traditional Chinese Medicine Systems Pharmacology Database
ADME	Absorption, Distribution, Metabolism, Excretion
BEAS-2B	Human Bronchial Epithelial Cell Line
EC_50_	Half-Maximal Effective Concentration
IC_50_	Half-Maximal Inhibitory Concentration
SI	Selectivity Index
CCL5	C-C Motif Chemokine Ligand 5
CCL3	C-C Motif Chemokine Ligand 3
ELISA	Enzyme-Linked Immunosorbent Assay
IL-6	Interleukin-6
GLP-1	Glucagon-Like Peptide-1
GIP	Gastric Inhibitory Polypeptide
HbA1c	Hemoglobin A1c (Glycated Hemoglobin)
MD	Molecular Dynamics
GMQE	Global Model Quality Estimation
QMEAN	Qualitative Model Energy Analysis
CYP2D6	Cytochrome P450 2D6
FASA	Fractional Accessible Surface Area
RMSD	Root-Mean-Square Deviation
Rg	Radius of Gyration
RMSF	Root-Mean-Square Fluctuation
Buried SASA	Buried Solvent-accessible Surface Area
TCID	Tissue Culture Infectious Dose
COVID-19	Coronavirus Disease 2019
OB	Oral Bioavailability
DL	Drug-Likeness
BBB	Blood–Brain Barrier
TPSA	Topological Polar Surface Area
UniProt	Universal Protein Resource
PDB	Protein Data Bank
RCSB	Research Collaboratory for Structural Bioinformatics
GROMACS	Groningen Machine for Chemical Simulations
GAFF	General Amber Force Field
AMBER	Assisted Model Building with Energy Refinement
TIP3P	Transferable Intermolecular Potential 3P
NPT	Isothermal-Isobaric
LINCS	Linear Constraint Solver
PME	Particle Mesh Ewald
VMD	Visual Molecular Dynamics
DMEM	Dulbecco’s Modified Eagle Medium
STR	Short Tandem Repeat
CCK-8	Cell Counting Kit-8
NVT	Canonical Ensemble
ANOVA	Analysis of Variance
